# Canine Multicentric Lymphoma: Diagnostic, Treatment, and Prognostic Insights

**DOI:** 10.3390/ani15030391

**Published:** 2025-01-30

**Authors:** Michelle do Carmo Pereira Rocha, Diana Araújo, Fátima Carvalho, Nuno Vale, Josiane Morais Pazzini, Marcus Antônio Rossi Feliciano, Andrigo Barboza De Nardi, Irina Amorim

**Affiliations:** 1 Department of Small Animal Clinic and Surgery, School of Agricultural and Veterinary Sciences, São Paulo State University (UNESP) “Júlio de Mesquita Filho”, Jaboticabal 01049-010, SP, Brazil; mc.rocha@unesp.br (M.d.C.P.R.); andrigobarboza@yahoo.com.br (A.B.D.N.); 2 Institute of Biomedical Sciences Abel Salazar (ICBAS), University of Porto (UP), Rua de Jorge Viterbo Ferreira 228, 4050-313 Porto, Portugal; dianac.araujo@hotmail.com (D.A.); fatimafaria10@yahoo.com.br (F.C.); 3 PerMed Research Group, Center for Health Technology and Services Research (CINTESIS), Rua Doutor Plácido da Costa, 4200-450 Porto, Portugal; nunovale@med.up.pt; 4 CINTESIS@RISE, Faculty of Medicine, University of Porto, 4200-319 Porto, Portugal; 5 Department of Community Medicine, Information and Health Decision Sciences (MEDCIDS), Faculty of Medicine, University of Porto, Rua Doutor Plácido da Costa, 4200-450 Porto, Portugal; 6 Clinic My Pet—UniSALESIANO, Araçatuba 16050-130, SP, Brazil; josipazzini@hotmail.com; 7 Department of Veterinary Medicine, University of São Paulo, Pirassununga 13635-900, SP, Brazil; marcusfeliciano@usp.br; 8 Institute of Molecular Pathology and Immunology, University of Porto (IPATIMUP), Rua Júlio Amaral de Carvalho, 45, 4200-135 Porto, Portugal; 9 Institute for Research and Innovation in Health (i3S), University of Porto, Rua Alfredo Allen, 208, 4200-135 Porto, Portugal

**Keywords:** dog, lymphoma, chemotherapy, immunotherapy, comparative oncology

## Abstract

Lymphoma accounts for 24% of all documented canine neoplasms and 85% of hematological malignancies, while multicentric lymphoma corresponds to 84% of all canine lymphomas. Canine lymphomas of B-cell origin account for 60% to 80% of lymphomas. Similar to humans, the histologic grade, architecture, as well as immunophenotype determination, are crucial. These lesions are the most prevalent spontaneous tumors in dogs and this species may be a valuable animal model for the study of human non-Hodgkin’s lymphoma. Therefore, it is important to investigate and assess therapeutic responses and to seek predictive and prognostic factors in order to allow for the development of an individualized and more effective therapy that increases survival. This review aims to describe current knowledge on the diagnosis, treatment, and prognostic factors of canine multicentric lymphoma.

## Simple Summary

Lymphoma is one of the most prevalent malignancies in dogs. It is known that this disease shares similarities with human non-Hodgkin’s lymphoma. For this reason, dogs may be an important animal model for studying this neoplasm. Thus, assessments of therapeutic responses and studies of the predictive and prognostic factors contributing towards an efficacious therapy are crucial. This review explores multicentric lymphoma diagnosis, treatment, and prognostic insights based on comparative oncology in canines.

## 1. Introduction

Lymphomas encompass a diverse group of tumors that originate from lymphoreticular cells. They can develop in practically any body tissue, but typically affect lymphoid tissues such as the lymph nodes, spleen, and bone marrow [1,2].

In dogs, lymphoma accounts for 24% of all documented neoplasms and 85% of diagnosed hematological malignancies [3]. They are classified according to their anatomical location, histologic characteristics, and immunophenotype; the most prevalent forms are: multicentric, gastrointestinal (GI), mediastinal, and cutaneous [1,2].

Multicentric lymphoma corresponds to 84% of all CL [1,2], ranging from 53–83% [4,5], and usually displays a superficial, painless lymphadenopathy [1,2,5].

These neoplasms occur due to clonal expansion of lymphoid cells that exhibit specific morphologic and immunophenotypic characteristics [1,2]. Similar to humans, B-cell CL accounts for 60–80% [1,3]; T-cell CL corresponds to 10–38%; mixed B and T-cell lymphomas represent 22%; and null cell tumors (neither B nor T-cell immunoreactive) account for less than 5% of CLs [1].

The determination of lymphoma histologic grade is based on the number of mitoses per high power field (low grade: 0–5 mitoses/400×; medium grade: 6–10 mitoses/400×; high grade: ≥10 mitoses/400×); the architecture, growth pattern (diffuse or follicular) and immunophenotype [1,6,7].

Since lymphomas are the most prevalent spontaneous hematopoietic tumors in dogs [8,9], this species may be a valuable and comparable animal model for human non-Hodgkin lymphoma (NHL) research [8,10], despite a lower occurrence of high-grade lymphomas in the latter [8].

The continuous progress in animal cancer diagnosis and treatment [9] favors the development of more effective and individualized therapies that aim to increase the patient’s quality of life. This review aims to describe the state of the art regarding the diagnosis and treatment of canine multicentric lymphoma (CML) from the comparative pathology point of view.

## 2. Clinical Signs

**Figure 1:**
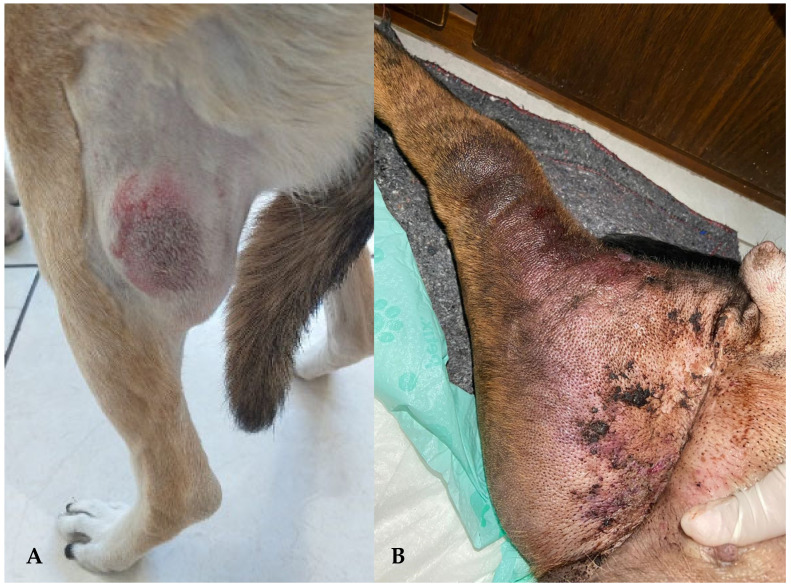
(**A**) Mixed-breed female dog of 7 years old, diagnosed with diffuse large B-cell lymphoma (DLBCL) through aspiration cytology of the left popliteal lymph node; and (**B**) mixed-breed male dog of 10 years old, diagnosed with immunoblastic B-cell lymphoma presenting left hind limb edema.

Canine lymphoma may display different clinical presentations. The majority of animals present generalized lymphadenopathy (Figure 1), mainly affecting mandibular and prescapular lymph nodes, liver and spleen enlargement, and bone marrow involvement [1,2].

Nonspecific clinical signs like anorexia, weight loss, vomiting, diarrhea, emaciation, ascites, dyspnea and fever, can manifest [1,2]. Dogs diagnosed with T-cell lymphoma more likely exhibit systemic clinical signs, such as anemia, polyuria, and polydipsia, mainly due to the hypercalcemia that is more usually associated with this immunophenotype (i.e., substage b) [11,12]. Additionally, a recent study found a correlation between proteinuria and advanced stages of this disease; this presentation is significantly negatively associated with the survival time in dogs newly diagnosed with lymphoma [11].

Anemia is also a known paraneoplastic syndrome of lymphoma and is directly related to a worse prognosis of the disease, with the reported prevalence ranging from 30–77%. Studies have suggested that anemia, in combination with the detection on blood smears of morphological anomalies in ≥3 red blood cells, particularly eccentrocytes, reinforces the clinical suspicion of lymphoma. The mechanism of its occurrence is correlated with the chronic disease inflammation that will cause oxidative stress, thus decreasing the bioavailability of iron [13]. However, it is important to highlight that anemia can also be caused by the infiltration of neoplastic lymphocytes in the patient’s bone marrow, which characterizes stage V of the disease and leads to a drastic limitation of normal red blood cell regeneration [1,13].

## 3. Diagnosis

### 3.1. Clinical Pathology

When lymphoma is suspected, the diagnosis involves a physical examination, a complete blood count (CBC) that includes a platelet count, and a serum biochemical profile (containing serum total or ionized calcium) [1,2]. Most animals exhibit mild-to-moderate non-regenerative anemia, although this may also result from blood loss (as observed in GI lymphoma) or immune-mediated hemolytic anemia. Anemia and neutrophilia are more frequently associated with T lymphomas [14,15]. Frequently, mild and asymptomatic thrombocytopenia is observed, and, sporadically, thrombocytosis. Most animals exhibit mild abnormalities in their hemostatic profile, indicative of hypercoagulability, which often continues throughout chemotherapy. Proteinuria is commonly identified in dogs with this condition [16].

The presence of hypercalcemia is almost exclusively associated with TCL and has been associated with a more aggressive behavior of this condition, which can lead to a serious electrolyte imbalance, with consequent cardiac, gastrointestinal, and renal disorders such as polyuria and polydipsia [14,15].

Nevertheless, for a conclusive diagnosis, a microscopic evaluation is essential, allowing for subsequent histological classification, lesion grading, and immunophenotyping [1,2].

### 3.2. Microscopy (Cytology, Histopathology Classification and Grading)

Cytological examination with a fine-needle aspiration (FNA) from a neoplastic lymph node or mass is a rapid, sensitive, and minimally invasive technique for diagnosing high-grade CL, and is the preferred diagnostic method [17,18]. However, cytology may be inadequate for diagnosing low-grade CL or for characterizing atypical lymphoid proliferations. A histological examination enhances the diagnosis of low-grade CL and facilitates further disease subclassifications [16].

**Figure 2:**
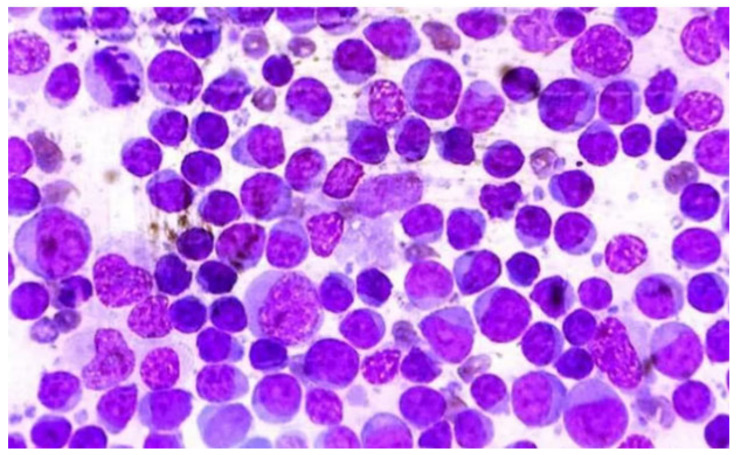
Cytology of popliteal lymph node of male Rottweiler dog, 10 years old, diagnosed with a DLCBL (OMS). Note the high cellularity of lymphocytes and the presence of lymphoglandular corpuscles. (40× magnification, panopticon stain). Kindly provided by Vetmol-SP, Brazil and all rights reserved.

Usually, neoplastic cells are large, lymphoid cells (more than twice the diameter of a red blood cell or larger than neutrophils). These might display visible nucleoli and basophilic cytoplasm, or fine chromatin with indistinct nucleoli [1] (Figure 2).

**Figure 3:**
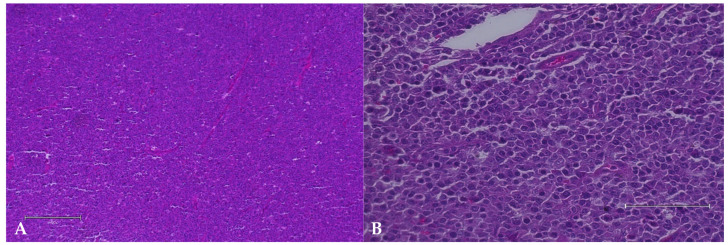
Histopathology of the popliteal lymph node of a mixed breed dog, 8 years old, diagnosed with immunoblastic B-cell lymphoma: (**A**) microscopically, loss of the follicular lymph node architecture is seen, which is now replaced by a uniform population of round neoplastic cells. (HE, 40×, Bar: 50 µm); and (**B**) at a higher magnification, the lesion is composed of large neoplastic lymphocytes, exhibiting nuclear pleomorphism and multiple nucleoli (HE, 200×, Bar: 100 µm).

Histologically, CL is defined according to several morphological criteria, as follows: growth pattern; nuclear size; nuclear morphology; chromatin pattern; number and location of nucleoli; mitotic index; and immunophenotype [16]. Typically, capsular disruption and replacement of the normal nodal architecture by neoplastic lymphocytes are observed [1] (Figure 3).

**Figure 4:**
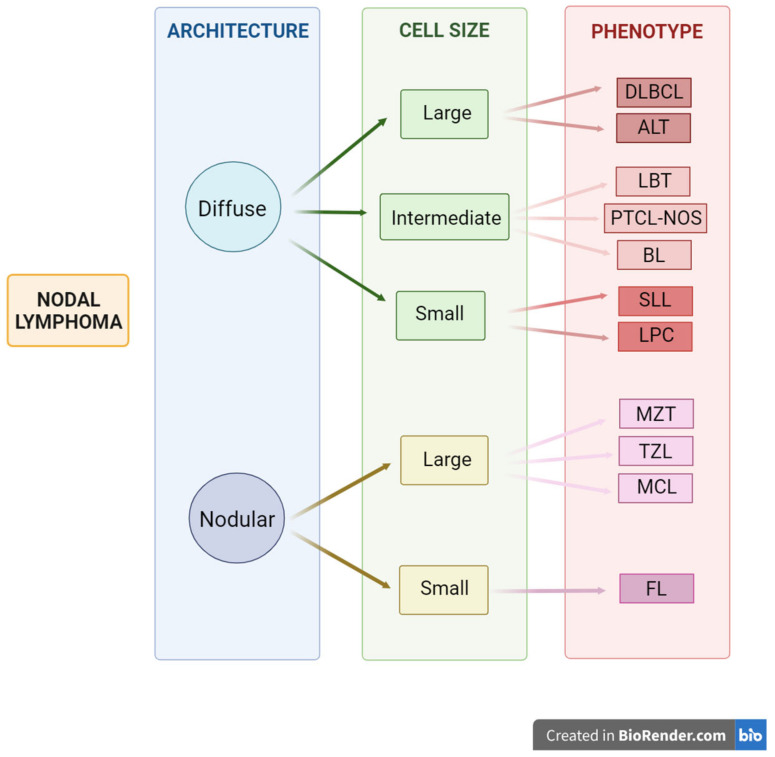
Schematic representation of the criteria used for the histological classification of canine nodal lymphoma. Created with BioRender.com accessed on 27 September 2024.

Several histological subtypes are recognized, and various human classification systems have been adapted to CL, such as the WHO and Kiel classifications and the NCI Working Formulation [1,2] (Table 1 and Figure 4).

The CL histological grade determination categorization as low, intermediate, or high, and the tissue architecture evaluation, as either diffuse or follicular (Figure 4) yield valuable information [1]. Valli et al. (2011) examined the growth pattern, the relationship between cellular nodules and remnants of non-neoplastic follicles, nuclear size, nuclear morphology, the number of mitoses per high field, and the immunophenotype. Nuclear size is considered small (<1.5 times the size of a red blood cell); intermediate, (1.5–2× the size of a red blood cell); or large (if >2× the size of a red blood cell). Regarding mitoses, lymphomas presenting 0–5 mitoses/400× are graded as 1 or low-grade; those with 6–10 mitoses/400× are considered grade 2 or medium-grade; and those exhibiting more than 10 mitoses/400×, are graded as 3 or high-grade [6].

### 3.3. Immunohistochemistry

Immunohistochemical studies are currently essential to complement the histological diagnosis and to provide other valuable diagnostic, prognostic, or even predictive information [1,19].

**Figure 5:**
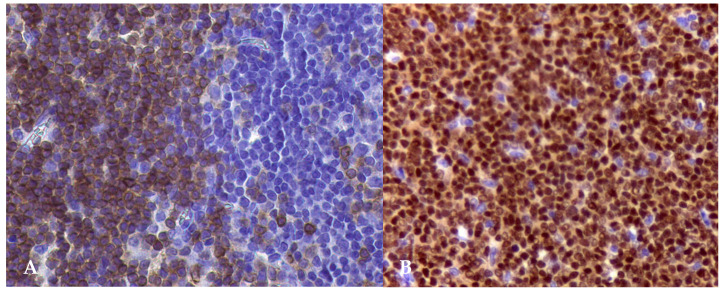
(**A**) Canine inguinal lymph node of a Labrador retriever, 6 years old, diagnosed with peripheral T-cell lymphoma. IHC for CD3 antibody counterstained with Mayer’s hematoxylin. The neoplastic cells present strong CD3 immunoexpression (40×); and (**B**) popliteal lymph node of a male Rottweiler, 10 years old, diagnosed with diffuse large B-cell lymphoma. IHC for PAX5 antibody counterstained with Mayer’s hematoxylin. The neoplastic cells present strong CD3 immunoexpression (40×). Kindly provided by Vetmol-SP, Brazil and all rights reserved.

To precisely ascertain the immunophenotype, antibodies specific to lymphocyte markers are used. T-cell markers include CD3 (pan T), CD4 (helper T), and CD8 (cytotoxic T), while B-cell markers encompass CD79a, CD20, and CD21 (Figure 5). The antibodies against these molecules may also have potential therapeutic value if the tumor cells can be used as targets by these antibodies [1].

The programmed death 1 (PD-1)/PD ligand 1 (PD-L1) pathway serves as an essential checkpoint, playing a key role in controlling immune responses driven by T cells. PD-L1 expression can be assessed through immunohistochemistry (IHC) on both tumor cells and peritumoral immune cells in various human and canine cancers, including lymphomas [20,21]. For PD-L1 assessment, the specific membranous immunoreactivity pattern is often considered; however, statistically significant prognostic value was found in investigations that evaluated the total stained area and that took into account cytoplasm immunolabelling [21,22]. In addition, others have evaluated the expression of PD-L1 using different techniques that can provide additional and complementary information to IHC such as flow cytometry and quantitative polymerase chain reaction (RT-qPCR) [23].

### 3.4. Polymerase Chain Reaction (PCR)

Since a lymphoma diagnosis based on cytologic and histologic features is not always possible, advanced molecular techniques may be fundamental to obtain a final diagnosis and for further tumor characterization. Various methods can analyze tissues and cells obtained from peripheral blood, lymph nodes, non-lymphoid sites, and effusions, and may encompass polymerase chain reaction (PCR) techniques [1].

Clonality is a hallmark of malignancy, implying that the malignant cell population theoretically originated from the proliferation of a single, malignant clone characterized by a specific DNA region unique to that tumor. It is expected that, in a T-cell lymphoma, all malignant cells should possess the same DNA sequence for the variable region of the T-cell receptor gene. In a B-cell lymphoma, the tumor cells should exhibit similar DNA sequences in the variable region of the immunoglobulin (Ig) receptor gene [1]. The most-used technique is the PCR assay for antigen receptor rearrangement (PARR), which aims to amplify the variable regions of the T-cell and Ig receptor genes for the detection of clonal lymphocyte populations in dogs [1,16].

Other PCR-based techniques are being developed, consisting of the evaluation of microRNAs (miRNAs) expression in the lymph nodes and serum of CL [24,25,26]. Indeed, some miRNAs are associated with immunophenotypes and clinical CML outcomes. Specifically increased expression of the miR-17–92 cluster, the miR-29 family, and miR34a present a strong association with B-cell lymphoma, while upregulation of the miR-181 family is associated with T-cell lymphoma. These miRNAs may be valuable for lymphoma diagnosis and in predicting the immunophenotype from clinical lymph node samples. MiR-19a, miR-19b, and miR-148 exhibited no overlapping lymph node expression between healthy dogs and those with B-cell lymphoma, indicating their potential as effective predictive biomarkers. At the time of relapse, very few miRNAs with altered expression persisted compared to the initial diagnosis. The upregulation of miR-127 in lymph nodes was observed exclusively in dogs with B-cell lymphoma during relapse, suggesting a potential association with chemoresistance or chemotherapy induction. Lastly, upregulation of the miR-181 family and downregulation of miR-29b and miR-150 were related with a poor response to therapy and survival for B-cell lymphoma, while an increased expression of miR-155 and miR-222 were negatively associated with outcomes in both B- and T-cell lymphoma [26].

Molecular techniques may be used to detect early recurrence and to more accurately define the clinical stage, allowing for the determination of the so-called “molecular remission rate” [1].

### 3.5. Clinical Staging

Once the diagnosis is confirmed, it remains essential to assess the extent of the disease and to establish the clinical stage of the condition [1]. The WHO proposes guidelines for CML staging (Table 2); the determination of the respective substages requires a comprehensive patient history, a complete physical examination (stages I–IV) and an evaluation of the peripheral blood and bone marrow (stage V) [16]. More than 80% of dogs are diagnosed at advanced stages (III-V) [1,9].

### 3.6. Diagnostic Imaging

Roughly 60–75% of CML patients exhibit thoracic radiograph abnormalities, with 1/3 showing signs of pulmonary infiltrates and 2/3 showing thoracic lymphadenopathy, along with widening of the cranial mediastinum. Clinical presentations may also include blood abnormalities or paraneoplastic signs [1,2,27]. About 27–34% of cases have diffuse pulmonary infiltration detected through X-rays [1,2]. In half of the cases, abdominal radiographs reveal medial iliac and/or mesenteric lymph node involvement, and spleen or liver involvement [1].

**Figure 6:**
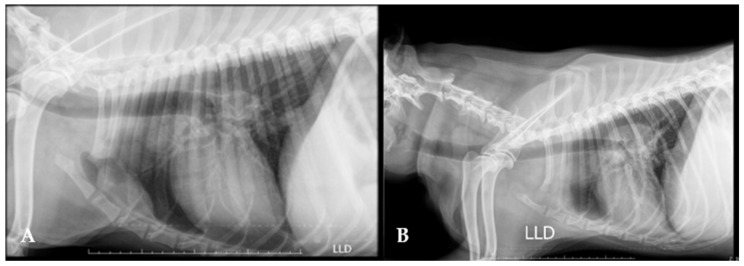
(**A**) Lateral thoracic radiograph of a 5-year-old mongrel dog with multicentric lymphoma and sternal lymph node involvement; and (**B**) thoracic radiograph of an 8-year-old shih tzu dog with pulmonary edema and cranial pleural effusion due to multicentric lymphoma.

For standard cases, imaging is primarily confined to thoracic radiographs, as there is no prognostic distinction between patients with stage III and IV disease (Figure 6). However, the presence of cranial mediastinal lymphadenopathy holds prognostic significance. If there are observable clinical signs linked to abdominal disease, additional imaging of the abdomen is necessary [1].

Imaging techniques have significantly developed, displaying greater sensitivity and specificity [28].

A recent study presented strain elastography combined with b-mode ultrasound as an affordable and less-invasive alternative to monitor therapeutic response as well as to predict early recurrence in dogs diagnosed with multicentric lymphoma. The research assessed 15 animals and showed that ultrasound can differentiate between benign and lymphomatous lymph nodes. The results showed significantly higher values of lymphomatous lymph nodes in the short-to-long axis ratio, elastography scales, and blue-to-green color histogram compared to normal lymph nodes. In addition, the lymph nodes affected before treatment had significantly higher values than those after treatment [29].

Unlike other tools, positron emission tomography (PET) is able to differentiate viable tumors from necrosis or fibrosis, in residual masses that may be present after therapy [30]. PET, using the glucose analog fluorine-18-fluorodeoxyglucose (18FDG), is widely employed in human cancer staging, enabling the detection of glucose metabolism related to malignant transformation before any anatomic changes [31]. In veterinary medicine, PET imaging is not frequently used since scanner availability and radioisotope access are limited. The use of 18FDG-PET in canine oncology has standard care potential; however, more data are required [31]. In three cases of CML, 18FDG-PET scans showed utility in pretreatment staging and in the assessment of treatment response imaging. It also identified lymph nodes and organs affected, highlighting the lesions’ post-chemotherapy resolution, proving to be an advantageous tool for lymph node involvement detection [31]. In CL, FDG-PET/CT provided a complete assessment of the disease’s extent and facilitated the cytologic diagnosis [32].

F-18-fluorothymidine (FLT), an in vivo marker of proliferative activity, has been shown to be better than FDG at distinguishing between indolent and aggressive lymphomas [33]. In CL patients, FLT-PET/CT was beneficial in disease site detection and in assessing both early and late chemotherapy responses. Additionally, FLT-PET/CT predicted early lymphoma recurrence in two patients prior to clinical detection, suggesting that FLT-PET is extremely sensitive [34]. These data may contribute to timing and intensity adjustments in chemotherapy protocols, resulting in survival improvements [34].

When compared to FDG-PET, it is unlikely that FLT-PET/CT is more advantageous at staging lymphomas, since there is a normally high FLT uptake in canine bone marrow. It is feasible that a significant modification in FLT uptake can be detected between normal dogs and those with bone marrow implication [34]. FLT-PET/CT is beneficial over FDG-PET in patients who have received chemotherapy or radiation therapy, since it is less likely to accumulate in inflammatory cells. Thus, FLT-PET and FDG-PET may be, in part, complementary [34].

In veterinary medicine, PET/CT imaging may constitute an important tool in remission status assessment, as tumor markers are rarely used to monitor the disease [34]. Its high cost may be worthwhile, given the possibility of obtaining a full-body image and discovering more sites of involvement [29].

## 4. Therapy

Several factors influence the therapeutic strategy, including, as follows: the disease’s stage and substage; the presence/absence of paraneoplastic disease; the patient’s overall physiological condition; the financial and time commitment of the owners; and their confidence level regarding the possible treatment-related success and/or side effects [1].

### 4.1. Chemotherapy

The reported survival time in untreated CL is 4–6 weeks after diagnosis [1]. As a systemic disease, chemotherapy is the elected treatment to achieve remission and prolong survival time [1,16,26]. Combination protocols are more efficient than single-agent ones, with systemic multiagent chemotherapy being the standard care treatment (Table 3) [1,35].

#### 4.1.1. First-Line Protocols

Several single-agent therapies are described, including a monotherapy with doxorubicin (DOX) [35,40,41,42], polyethylene glycol-(PEG)L-asparaginase [43,44], mitoxantrone [45] and lomustine (CCNU) [46]. Among these, the doxorubicin single-agent protocol stands out as the most effective but remains less effective than a multi-agent protocol with doxorubicin; it is a reasonable option if there are financial or time limitations and may be recommended for cases where treatment is primarily palliative [1,2,16,35].

A recent therapeutic advance for multicentric lymphoma was the development and approval of Tanovea^®^ (initially known as GS-9219, later VDC-1101, generically referred to rabacfosadine or RAB), a “double” prodrug with PMEG [9-(2-phosphonylmethoxyethyl) guanine]. It is currently the first and only fully FDA-approved treatment for canine lymphoma, offering an option for a different approach in the first rescue. Tanovea has shown effectiveness in treating various types of CL and relapsed canine B-cell lymphoma. It inhibits DNA polymerases α, δ and ε, resulting in DNA synthesis arrest and programmed cell death induction, and has an affinity for neoplastic lymphoid tissue, which limits its off-target toxicity [37].

Multi-agent protocols consist of a combination of cyclophosphamide, doxorubicin, vincristine, and prednisolone, referred to as CHOP, and often include L-asparaginase (L-CHOP). CHOP-based protocols yield the highest response rates and longest durations of response and. are used for the treatment of high-grade CL [1,16].

**Figure 7:**
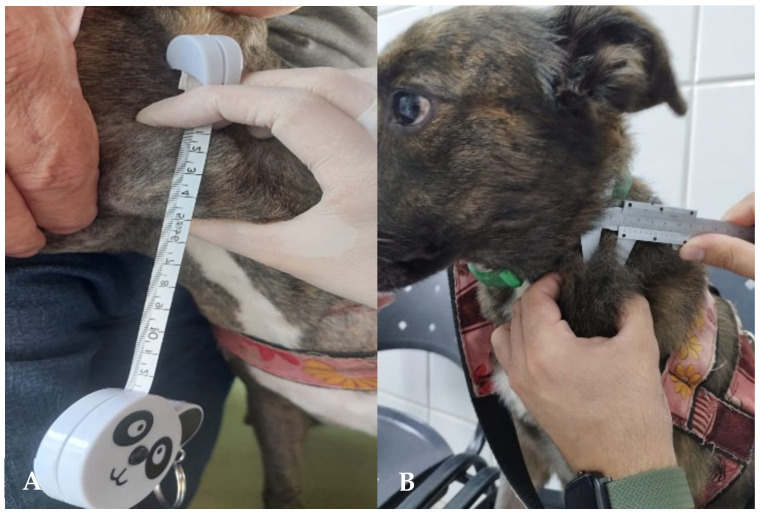
Dog diagnosed with DLCBL: (**A**) measurement of the affected ventral cervical lymph node at the time of diagnosis; and (**B**) measurement of the same lymph node at the tenth week of CHOP chemotherapy treatment. The patient was in complete remission.

Conventional CHOP-based regimens induce remission in 80–95% of dogs, with overall median survival times of 8–12 months; it is expected that 20–25% of patients undergoing these protocols survive for two years following the treatment initiation [1,2,3] (Figure 7).

In veterinary oncology, there is a pressing need for the individualization of antineoplastic chemotherapy protocols, due not only to the heterogeneity in the clinical behavior of neoplasms, but also the heterogeneity of patients, who may present with different ages, races, body scores and comorbidities that will directly impact the therapeutic response and tolerance to the chemotherapy protocol [35]. A prospective study used 30 dogs diagnosed with multicentric lymphoma that underwent a 15-week CHOP protocol, with the aim of determining the possibility of increased drug doses. The authors found that this would be feasible in 23 of the 30 dogs evaluated; of these, 18 had at least one of the protocol medications with an increased dose. Vincristine was successfully increased to 0.8 mg/m^2^ or higher in 11 dogs; cyclophosphamide to 300 mg/m^2^ or higher in 16 dogs; and doxorubicin to 35 mg/m^2^ or 1.4 mg/kg or higher in 9 dogs. The main side effect identified was neutropenia, which was the limiting factor for drug toxicity. The objective response rate was 100%. The median progression-free interval and the median overall survival time was 171 and 254 days, respectively [47].

**Figure 8:**
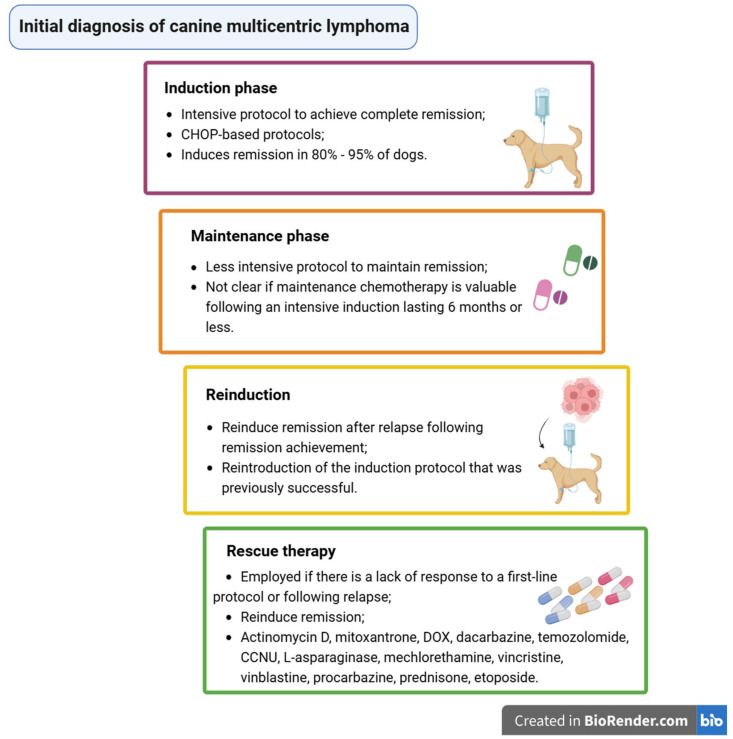
Chemotherapy phases in canine lymphoma treatment [1,2,3,16]. Created with BioRender.com accessed on 29 January 2025.

Chemotherapy is an intensive protocol aiming to achieve a complete remission (induction phase), followed by a less-intensive protocol to maintain remission (maintenance phase) [1,16]. If the patients relapse following remission, it is necessary to reinduce a remission (reinduction); ultimately, chemotherapy is used to induce remissions when cancer does not respond to induction and reinduction, using drugs not included in the initial protocols (rescue therapy) [1] (Figure 8).

Long-term maintenance chemotherapy is still questionable, since several dogs treated with CHOP-based protocols did not benefit from a maintenance phase after induction therapy [1,16]. After the first recurrence, reinduction using the initial induction protocol is recommended if the recurrence occurred at least 2 months after the end of the initial protocol [1].

Although the CHOP polychemotherapy protocol is considered the gold-standard treatment for canine high-grade lymphoma, there are some associated constraints that may eventually lead to treatment evasion, such as side effects in older patients or those related to possible comorbidities, the cost of therapy, and a need for the greater availability and commitment from the tutor. Therefore, one study evaluated the efficacy and prognostic value of the monotherapy treatment protocol, with lomustine and prednisolone acting as a first-line treatment in dogs with different histotypes of high-grade lymphoma. An overall response rate of 87% was achieved, with 15 patients achieving complete remission (50%) and 11 patients achieving partial remission (37%). A median survival time of 90 days was recorded, reinforcing the palliative utility of this protocol [48].

Another investigation evaluated the therapeutic efficacy of the recently approved drug rabacfosadine (RAB, Tanovea^®^) in combination with doxorubicin (DOX), in a population of 59 dogs with lymphoma. The protocol consisted of RAB (1.0 mg/kg IV) alternated with DOX (30 mg/m^2^ IV) every 21 days for up to six total treatments (3 cycles). The overall response was 93%, with a mean progression-free time of 199 days. The main side effect recorded was pulmonary fibrosis (six dogs). The authors concluded that this treatment approach is relatively safe and may be considered a reliable alternative to the actual conventional treatment of canine lymphoma [38].

Nevertheless, lymphoma is a disease that often displays several mechanisms that favor chemotherapy resistance; therefore, clinical signs recur, such as the mutation of the *MDR1* gene, which modifies the physiology of the P glycoprotein, thus hindering the action of chemotherapy molecules, especially the class of antitumor antibiotics, such as doxorubicin, the main component of the CHOP protocol [49]. In this context, a recent study based on the information that increased serum cortisol (COR) concentrations can induce glucocorticoid resistance by downregulating the glucocorticoid receptor (GCR), investigated the relationship between serum COR concentrations and the results of chemotherapy in dogs with lymphoma. It was shown that dogs presenting with high-COR concentrations exhibited a significantly lower response to chemotherapy; this group also presented a higher cellular expression of the GCR receptor than the group with lower COR concentrations. As such, it was suggested that the measurement of COR serum concentrations can serve as a potential prognostic factor and evaluation index [49].

#### 4.1.2. Rescue Protocols

Most dogs that achieve remission will eventually relapse or experience a disease recurrence, usually represented by the appearance of tumor clones or tumor stem cells that inherently own greater resistance to chemotherapy compared to the original tumor. These, commonly known as MDR clones, are either initially drug-resistant or acquire resistance following exposure to specific chemotherapy agents [1].

Other factors can lead to relapse such as inadequate dosing and frequency of chemotherapy administration, inability to achieve elevated concentrations of chemotherapeutic drugs in specific sites (like CNS), and initial treatment with prednisone alone [1].

Rescue protocols are employed when there is a lack of response to a first-line protocol or following relapse, and consist of both single or multi-agents that are usually not found in standard CHOP protocols [1,16]. However, a relapse after completing the first-line protocol allows for the inclusion of drugs used in the original therapy. The selection of a treatment protocol depends on the timing of relapse in relation to the first-line protocol, the drugs previously administered, and the clinician’s preferences. Rescue protocols usually reveal lower response rates, shorter response times (2–3 months), and more toxicity compared to first-line protocols [16]. The most common drugs used are actinomycin D, mitoxantrone, doxorubicin (if not used in the original induction protocol), dacarbazine (DTIC), temozolomide, CCNU, L-asparaginase, mechlorethamine, vincristine, vinblastine, procarbazine, prednisone, and etoposide (Table 4). In general, reported rescue response rates range from 40–90%, with median durations of 1.5 to 2.5 months, regardless of the protocol’s complexity [1].

A recent investigation attempted to evaluate the therapeutic response of chemotherapy protocols based on lomustine L-LOP (L-asparaginase, lomustine, vincristine and prednisolone) and L-LOPP (with the addition of procarbazine), currently considered to be excellent rescue protocol options in high-grade gastrointestinal (GI) and hepatosplenic (HS) canine lymphomas. Both protocols were well tolerated by patients, rarely triggering serious side effects [50]. The median progression-free survival for GI and HS lymphoma was 56 days (10 to 274 days) and 57 days (8 to 135 days), respectively; the median survival time for GI and HS lymphoma was 93 days (10 to 325 days) and 210 days (8 to 240 days), respectively [46].

A specific rescue protocol selection should consider the costs, time commitment, effectiveness, toxicity, and the clinician’s experience with the protocols. Nevertheless, employing diverse rescue protocols, changing as necessary depending on the response, will persist as long as clients are satisfied with their dog’s quality of life. Continuous administration of multiple rescue protocols can lead to several months of prolonged survival with a satisfactory quality of life [1].

#### 4.1.3. T-Cell Lymphoma Treatment

Approximately 10–38% of CMLs are of T-cell origin, of which the most common histological types are T-zone lymphomas, T-lymphoblastic lymphomas, and peripheral T-lymphomas. Of these, the latter two are considered high-grade neoplasms, presenting a poor prognosis and aggressive clinical behavior [1,16].

Currently, data regarding specific prognostic factors for TCL as an isolated clinical entity, are limited. The conditions related to a worse prognosis associated with the T immunophenotype are mainly the development of mechanisms of resistance to chemotherapy and the emergence of paraneoplastic syndromes [15]. In contrast with B-cell lymphomas, multicentric T-cell lymphomas exhibit similar initial response rates but significantly lower response durability, such as progression-free survival (PFS) following chemotherapy, including with CHOP-based protocols [1], carrying a poorer prognosis [16].

Alternative protocols have been suggested, like L-asparaginase-MOPP (mechlorethamine, oncovin, procarbazine, prednisone), which might exhibit a longer PFS and overall survival time (OST) [51]. A modified lomustine, vincristine, procarbazine and prednisolone (LOPP) protocol was used as a first-line treatment in dogs with T-cell lymphoma and demonstrated an improvement in median PFS (431 days) and in median survival time (507 days), suggesting its use as a first-line chemotherapy protocol [39]. Nevertheless, another investigation revealed comparable results between dogs with T- and B-cell lymphoma treated with the CHOP protocol [14].

Previous research evaluated multi-agent chemotherapy (vincristine, epirubicin and prednisolone) including either cyclophosphamide (CEOP) or lomustine (LEOP) as an initial therapy for canine high-grade T-cell lymphoma (HGTCL) and concluded that overall survival was improved for patients receiving LEOP compared to those receiving CEOP followed by lomustine-based rescue therapy. These results support further evaluation of lomustine as part of a first-line multi-agent therapy for patients with HGTCL [52].

#### 4.1.4. Drug Resistance

Given the restricted availability of alternative treatment choices for CL, the development of drug resistance significantly influences the prognosis [16].

Resistance to drugs may either be intrinsic to cancer cells or emerge subsequently to specific chemotherapeutic agents’ exposure [1]. It has been linked with the active removal of cytostatic drugs by transporter proteins of the adenosine triphosphate (ATP)-binding cassette (ABC) transporter superfamily such as the P-glycoprotein (P-pg) (ABCB1), MRP1 (ABCC1), and BCRP (ABCG2) [1,16]. Intrinsic drug resistance in CML was associated with increased mRNA expression of ABCB1 in B-cell lymphomas while T-cell lymphomas tended to be associated with ABCG2 overexpression [53].

To reverse drug resistance, P-gp and BCRP inhibitors or cytotoxic drugs that are neither P-gp nor BCRP substrates [16] might be useful. P-gp inhibitors, such as the tyrosine-kinase inhibitor masitinib, are potential candidates to treat drug resistance in CL [54].

Chemotherapeutic agents, such as alkylating agents and L-asparaginase, commonly employed in different rescue protocols, are not conventional substrates for the ABC transporters ABCB1 and ABCG2 [16].

### 4.2. Radiotherapy

Since it is a systemic disease, radiotherapy plays a restricted role in lymphoma. Its use has been advised for both localized and multicentric forms, as a mono- or adjuvant therapy [16]. Radiotherapy for CML involves radiating the entire body through a single session of whole-body irradiation (WBI) or through two distinct sessions of half-body irradiation (HBI). The last option is preferred due to reduced bone marrow and GI tract side effects. This can be executed either concurrently with chemotherapy or at the end of the protocol, and administered at either a high- or low-dose rate [16].

The high-dose rate protocol involves the administration of 8 Gray (Gy) on two consecutive days of 4 Gy doses each [16,55,56]. Some studies used this protocol, either during the CHOP-based chemotherapy protocol, with a median duration of first remission of 455 days and median OST of 560 days [55], or at the end of the CHOP-based chemotherapy protocol, reaching a median duration of 311 days in the first remission and a median OST of 486 days [56].

Another study used low-dose rate HBI of 6 Gy (8–14 cGy/min) during the CHOP-based protocol, achieving a first remission of 410 days and a median OST of 684 days [57].

### 4.3. Immunotherapy

Cancer therapies based on the immune system hold the potential to achieve prolonged and lasting remissions and may even present the opportunity for a cure [58].

#### 4.3.1. Monoclonal Antibodies

Monoclonal antibodies (mAbs) are a class of cancer therapeutic agents that promote the direct or indirect death of cancer cells. mAbs are able to change tumor cell signaling cascades or tumor–stroma interactions, mediate antitumor immune response through antibody-dependent cellular cytotoxicity (ADCC) and phagocytosis, and complement-dependent cytotoxicity (CDC) [59].

In individuals with non-Hodgkin’s B-cell lymphoma, initial remissions resulted from mAb-based therapies—R-CHOP protocols—in which “R” stands for rituximab, a recombinant chimeric murine/human antibody, specifically targeting the CD20 antigen. CD20 is a hydrophobic transmembrane protein in normal pre-B and mature B lymphocytes. After binding, rituximab induces a host cytotoxic immune response against CD20-positive cells [1]. Rituximab and other antibodies designed for human and mouse CD20 extracellular domains did not have success binding to canine CD20, despite the conservation of reported epitopes between both human and canine CD20 [60]. Therefore, immunotherapy application in CL is currently limited [61] and there is a scarcity of specifically designed mAbs for veterinary use; even fewer have been assessed in clinical trials involving companion animals [61].

#### 4.3.2. Adoptive T-Cell Transfer

Adoptive cell therapy (ACT) consists of a personalized cancer treatment approach involving the infusion of host immune cells with direct anti-cancer activity into the host with cancer. ACT holds several advantages. It relies on the in vivo development of a sufficient number of anti-tumor T cells with the necessary features for inducing cancer regression. A significant advantage of ACT is its ability to manipulate the host before cell transfer, creating a favorable microenvironment that enhances support for anti-tumor immunity; it is considered a “living therapy”, since the administered cells can proliferate in vivo and maintain their anticancer properties [62].

One study expanded T cells extracted ex vivo from dogs with lymphoma on artificial antigen-presenting cells in the presence of human interleukin (IL)-2 and IL-21. The autologous T cells were infused after CHOP chemotherapy, persisted for more than 49 days, and were trafficked to secondary lymphoid organs. The treatment improved survival, demonstrating the safety of autologous T-cell adoptive transfer in dogs [63].

CARs consist of artificial receptors made up of a single-chain antibody variable fragment design for a tumor antigen connected to an intracellular signaling domain and co-stimulatory molecules. CARs operate in a major histocompatibility complex (MHC)-independent manner, do not depend on patients’ antigen-presenting cells (APCs) for antigen presentation and are not required to be syngeneic to the patient’s immune system [64]. Current research focuses on the conception of canine CAR-T cells, specifically aimed at B-cell lymphomas and other cancer treatments [64].

#### 4.3.3. Oncolytic Virotherapy

Oncolytic virotherapy uses replication-competent viruses to eliminate cancer through the infection of tumor cells. This immunotherapeutic approach can stimulate de novo or improve the existing native immune responses [61].

A recombinant strain of the canine distemper virus (CDV) was able to infect CL cell lines in vitro and induce apoptosis. It also infected primary canine B- and T-cell lymphoma cells [65]. Another study investigated the oncolytic features of a strain of Newcastle disease virus in reducing cell viability in humans and CL cells [66]. In vitro studies demonstrated that reovirus, a powerful oncolytic virus, induced apoptosis and decreased the viability of CL cells [67].

#### 4.3.4. Immunomodulators

Cytokine therapy purpose is to improve immune responses and tumor control in different spontaneous cancers [61].

A phase I study assessed the safety of large multivalent immunogens (LMIs) formulated using autologous lymphoma cell membranes combined with IL-2 and granulocyte-macrophage colony-stimulating factor (GM-CSF) in dogs with untreated B-cell lymphoma. The results revealed no significant toxicity, no side effects in the disease-free interval and that half of the canine patients exhibited measurable delayed-type hypersensitivity reactions to intradermal LMI, which indicates specific cell-mediated immunity [68].

#### 4.3.5. Vaccines

Various strategies for antitumor vaccines have been employed in dogs diagnosed with lymphoma [1]. Some investigations used lymphoma cell extracts combined with Freund’s adjuvant as a cancer vaccine approach. Although initial data reported a treatment benefit [69], this was later assigned to the use of the Freund’s adjuvant [70].

Other studies used an intralymphatic administration of an autologous tumor vaccine following induction with chemotherapy; however, the results were inconsistent [71,72,73].

Autologous CD40-activated B-cells loaded with total RNA derived from autologous lymphoma cells were administered as an adjuvant to canine patients with lymphoma after achieving a complete response with chemotherapy. This vaccination induced a functional tumor-specific T-cell response in vivo; however, there was no improvement in treatment outcomes following the first-line treatment, only an enhancement in the rescue therapy results [74].

A DNA vaccine that targeted canine telomerase reverse transcriptase (cTERT) triggered an immune response against telomerase in dogs with lymphoma [75]. The concurrent application of the vaccine and COP chemotherapy protocol not only sustained the immune response but also led to an extended survival, without notable adverse events, in dogs with B-cell lymphoma [76]. A genetic vaccine that targeted dog telomerase (dTERT) based on adenovirus (Ad)/DNA electro-gene-transfer (DNA–EGT) technology, Tel-eVax, was combined with CHOP and was shown to be safe and immunogenic, exhibiting a significant influence on DLBCL canine patients’ survival [77].

An autologous vaccine including hydroxyapatite ceramic powder with autologous heat shock proteins (HSP) purified from a neoplastic lymph node demonstrated efficacy in disease control, extending overall survival and time to progression in canine DLBCL and multicentric indolent B-cell neoplasia, without increasing toxicity [78,79]. In order to assess the safety and efficacy of this last vaccine, APAVAC^®^, a retrospective study included all dogs treated with chemo-immunotherapy to date and compared them with those treated with chemotherapy only. The canine patients who received the vaccine protocol had improved outcomes, regardless of the histotype and evaluated prognostic factors, demonstrating great tolerability to the vaccine [80].

#### 4.3.6. Immune Checkpoint Blockage

Immune checkpoint inhibitors (ICIs) play a role in stimulating anti-tumor immune responses by disrupting co-inhibitory signaling pathways, thereby facilitating the immune-mediated elimination of tumor cells [81].

In humans, ICIs targeting cytotoxic T-lymphocyte associated protein 4 (CTLA-4) and the programmed death 1 (PD-1)/PD ligand 1(PD-L1) axis have demonstrated clinical activity in several cancers [82]. PD-L1 is increased in canine B-cell lymphomas when compared to normal B cells. Regarding tumor cells from T-cell lymphoma and normal T cells, both exhibited low to negative expressions of PD-1 and PD-L1. Furthermore, tumor-infiltrating lymphocytes from B-cell and T-cell lymphomas displayed an increased expression of both PD-1 and PD-L1 when compared to B and T cells from healthy dogs [83]. Chemotherapy-resistant canine B- and T-cell lymphoma cell lines showed increases in PD-1 and PD-L1 expression in vitro, compared to non-chemotherapy tumor cells [83].

In addition, PD-1 expression is upregulated on both peripheral and tumor-infiltrating T cells. An upregulation expression of CTLA-4 on CD4+ T cells was found in the peripheral blood of dogs with B-cell high-grade lymphoma and was related with a poor prognosis [84].

Therefore, CLs display upregulated checkpoint molecule expression, potentially representing new therapeutic targets and prognostic factors [83,84].

Other immune checkpoints, such as CD47 and verdinexor, were studied. The CD47/SIRPα axis consists of an immune checkpoint that controls macrophage activation. An in vitro and in vivo study assessed the combination of CD47-blockade with 1E4-cIgGB, a canine-specific antibody to CD20. The results demonstrated that 1E4-cIgGB, as a single agent, was able to induce a therapeutic response against CL in vivo. The combination with CD47-blocking therapies exhibited enhanced responses, showing synergy both in vitro and in vivo, achieving cures in all mice bearing CL [85].

KPT-335 (verdinexor) is an orally bioavailable selective inhibitor of nuclear export (SINE) that demonstrated anti-tumor effects in a previous phase I study on NHL. A phase II study was conducted to evaluate efficacy and safety in a broader population of dogs with lymphoma. The oral single-agent administration of KPT-335 was well-tolerated and resulted in a 37% objective response rate (ORR), with T-cell lymphoma patients experiencing an ORR of 71% [86].

## 5. Prognosis

The CL prognosis is extremely variable and depends on several factors, such as clinical evidence, pretreatment clinical results, histology, immunophenotype, grade, proliferation markers, molecular prognosticators, and biomarkers [1,16] (Table 5). Although the curable rate is low (<10% of cases), complete response and good quality of life during prolonged remissions and survival are common [1].

The two most reliable prognostic factors are immunophenotype and WHO substage. Dogs with CD3-immunoreactive tumors exhibit significantly shorter remission and survival times and those with B-cell lymphomas with lower B5 antigen levels (found in 95% of nonneoplastic lymphocytes) also have shorter remission and survival times. Low levels of class II MHC expression on B-cell lymphomas are predictive of poor outcomes. Dogs diagnosed with WHO substage *b* disease do poorly compared to dogs with disease substage *a*, and those in stages I and II have a more favorable prognosis than those in more advanced stages [1].

Dogs with intermediate or high-grade (large cell, centroblastic, and immunoblastic) lymphomas usually respond to chemotherapy but can relapse early; those with low-grade lymphomas (small lymphocytic or centrocytic) have a poorer response to chemotherapy but exhibit improved survival [1].

T-cell lymphomas mostly exhibit shorter remission and survival times compared to B-cell lymphomas [87,88,89].

Most T-cell lymphomas are CD4+/CD45+ high-grade lymphomas (lymphoblastic or peripheral T-cell lymphomas) expressing low II MHC with an aggressive disease course, regardless of the treatment regimen (median survival of 159 days). A minority of T-cell lymphomas are CD4+/CD45−, expressing high class II MHC and apparently show a more indolent disease course [90].

Assays evaluating proliferation, including the analysis of bromodeoxyuridine (BrdU) uptake, reactivity to Ki67 antibody, and argyrophilic nucleolar organizer region (AgNOR) indices, have been used to assess the proliferative activity of tumor cells in dogs undergoing combination chemotherapy, providing prognostic information [1].

**Figure 9:**
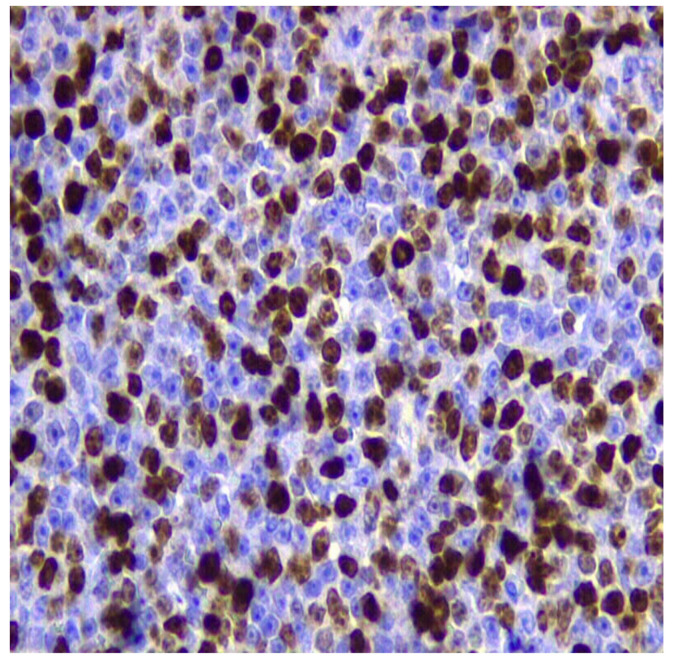
Inguinal lymph node of a Labrador retriever, 6 years old diagnosed with multicentric BLBCL lymphoma. The neoplastic population reveal specific nuclear Ki67 immunoreactivity. IHC for Ki67 counterstained with Mayer’s hematoxylin (400×). Kindly provided by Vetmol-SP, Brazil and all rights reserved.

Studies explored the prognostic significance of ki67 assessed by flow cytometry and IHC in dogs with B-cell lymphoma treated with the CHOP protocol and have demonstrated a correlation between Ki67 and survival time. Thus, the results indicate that Ki67-low is a good prognostic marker [91,92] (Figure 9).

Regarding molecular biology, the most common subtypes of CL (DLBCL, BL, MZL, LBT, PTCL, and TZL) can be subdivided into three molecular subgroups that have prognostic significance, such as high-grade T-cell lymphomas (LBT, PTCL), low-grade T-cell lymphomas (TZL), and B-cell lymphomas (DLBCL, BL, and MZL). TZLs have been reported to exhibit a slow and indolent progression and might initially respond well to a conservative approach or low-intensity chemotherapy, aiming to minimize the risk of treatment-related toxicity with a low possibility of accelerated tumor progression. In contrast, high-grade LBTs and PTCLs are characterized by aggressiveness and rapid progression, responding poorly to conventional chemotherapy [93].

Biomarkers consist of serum proteins that can be used for diagnosis and/or disease monitoring. In CL, acute phase proteins, such as thymidine kinase 1 (TK1), monocyte chemotactic protein-1 (MCP-1), vascular endothelial growth factor (VEGF), matrix metalloproteinase (MMP) and endostatin have been assessed [16]. Increased serum MCP-1 in dogs with lymphoma treated with CHOP-based protocols is associated with a decreased disease-free interval [96]. Only serum TK1 levels displayed prognostic value, since these can be used as a tumor marker for prognosis and also for relapse prediction and the prior recurrence of clinically detectable disease in dogs with lymphoma undergoing chemotherapy [94].

TK1, involved in DNA precursor synthesis, is used as a serum biomarker in cancer diagnostics in both human and veterinary medicine. A study quantified the concentration of TK1p through the ELISA test in the serum of 51 dogs with lymphoma and 149 healthy individuals in order to compare these data and identify correlations with disease prognosis and also monitor the therapeutic response of these patients. The study showed that the mean TK1p in the sera of dogs with tumors was significantly higher than that in healthy dogs. In addition, the test had high prognostic value and sensitivity for the disease, suggesting that the combination TK1 + CRP (C-reactive protein) may be useful in a panel of biomarkers to monitor the disease throughout and after chemotherapy treatment [95].

Furthermore, a recent genetic assay for hematopoietic neoplasms in dogs, known as the K9 assay, followed more than 31 genes designed for the genomic profile of this neoplasm. This study, which included several samples of different histological types of B-cell and T-cell lymphomas, concluded that the *TRAF3* gene was the most frequently mutated gene in B-cell lymphoma subtypes, with adverse prognostic value for this histotype. The correlation of the *SETD2* gene mutation with a shorter time to disease recurrence was also highlighted. Finally, in T-cell lymphomas, considered to be those with the most aggressive behavior, the *SATB1* and *FBXW7* genes were frequently mutated. *SATB1* was the most important mutation identified in these histotypes, being responsible for the global transcription regulator and chromatin organizer. Its silencing leads to impaired T-cell development and function, which indicates that *SATB1* is an intriguing target for further exploration in veterinary medicine. *FBXW7* has been identified as a tumor suppressor gene and is correlated with a worse prognosis in T-cell lymphomas [103].

In various diseases, cell-free DNA (cfDNA), specifically in the form of nucleosomes, is released into circulation due to apoptosis and necrosis. Nucleosomes are small chromosomal segments consisting of DNA wrapped around a histone core, forming an octamer structure; however, this field remains relatively underexplored as a tumor biomarker in canine oncology. One study quantified nucleosome levels in dogs with different cancers, revealing that those with cancer display a 49.8% sensitivity and 97% specificity in increased nucleosome detection, with lymphoma among the most commonly detected malignancies [97].

In order to determine if nucleosome levels could be used to distinguish between healthy dogs and those with lymphoma, researchers, using the Nu.Q™ H3.1 assay, identified an approximately sevenfold increase in plasma nucleosome levels in dogs with lymphoma compared to healthy controls, with significant elevations noted, particularly in B-cell lymphomas and at more advanced stages [99].

Investigating cfDNA as a diagnostic tool, another study demonstrated that dogs with neoplasia had significantly higher cfDNA levels than non-neoplastic and healthy dogs, with higher cfDNA linked to shorter survival times. In fact, a significant correlation between lymph node diameter and cfDNA concentrations in dogs with multicentric lymphoma was reported. Thus, cfDNA may serve as a potential indicator of treatment response in lymphoma [98].

Additionally, nucleosome levels appear to be valuable for monitoring disease progression, with plasma concentrations markedly decreasing during remission and rising again upon relapse, correlating with disease state [100].

Nucleosome levels appear to be an effective tool with which to distinguish healthy dogs from those with cancer, suggesting their potential as diagnostic markers [99]. Furthermore, plasma nucleosome measurement may serve as a reliable marker with which to evaluate treatment response and disease advancement in hematopoietic malignancies in dogs [100].

White blood cell parameters have been explored as prognostic markers in human DLBCL such as absolute monocyte levels and ratios of lymphocyte-to-monocyte (LMR), neutrophil-to-lymphocyte (NLR), and platelet-to-lymphocyte (PLR), whether assessed individually or in combination [104,105,106,107]. Increased neutrophil and monocyte counts are related to a poorer prognosis in dogs with multicentric lymphoma treated with CHOP-based chemotherapy protocols [96,101]. NLR and LMR ratios have been identified as predictors of time to progression, progression-free survival, lymphoma-specific survival, and overall survival in dogs with DLBCL undergoing CHOP-based chemotherapy [108,109]. Another investigation assessed the prognostic value of hematological ratios (NLR, LMR, PLR and platelet-to-neutrophil—PNR) for time to progression and lymphoma-specific survival in dogs with DLBCL treated with a 19-week CHOP chemotherapy protocol, demonstrating that PNR is an independent prognostic marker for the time to progression rate, being a predictor of early lymphoma progression [102].

## 6. Conclusions

Lymphoma is one of the most extensively studied malignancies in veterinary medicine; however, many challenges remain. The ongoing advancement of oncological treatments introduces new challenges such as the development of drug resistance and treatment-related toxicities. Moreover, the identification of biomarkers potentially improves early diagnosis and the monitoring of treatment response. Future research must focus on developing approaches to extend survival and prioritize the quality of life.

## Figures and Tables

**Table 1:** Classification systems for canine lymphoma. Adapted from Vezzali et al., 2010 [8].

Cell Type	WHO Classification		Working Formulation	Kiel Classification
B	Precursor Mature	B-ALL/B-LBL	ALL, LBL	Lb
		B-CLL/B-SLL	B-CLL, B-SLL	Lc
		LLI	-	-
		LPL	SLLP	PI
	Follicular	MCL	DSCCL	Cc
		FCCL-I	FSCCL	Cc
		FCCL-II	FMCL	Cc-Cb
		FCCL-III	FLCL	Cb
		NMZ	CLL, FSCCL, DSCCL, DMCL	Lc, Cc, Cb
		SMZ		
		MALT-L		
	Plasmocytic	PCT Indolent	Extramedullary plasmacytoma	Extramedullary plasmacytoma
		PCT Anaplastic Myeloma	Multiple Myeloma	Multiple Myeloma
	B-LCL	DLBCL, DLBCCL	DMCL, DLCL, DLCCL	Cc, Cb
		LCIBL	LCIBL	Ib
		T-cell rich B-LCL	DMCL, DLCL	Cb
		Thymic B-LCL	DMCL, DLCL	Cb
	Burkitt-type, Burkitt-like	SNCCL	-	-
T	Precursor	T-ALL/T-LBL	ALL, LBL	Lb
	Mature	T-CLL/T-SLL	CLL, SLL	Lc
		LGL	-	-
		NK-CLL	-	-
	Cutaneous	CEL	MF/SS or DLCL, DMCL, DSCCL	MF/SS
		CNEL	MF/SS-like or DLCL, DMCL, DSCCL	MF/SS-like
	PTCL	-	DSCCL, DMCL, DLCL, DLCCL	-
	ATL	ACL	DLCCL, DMCL, DLCL, DLCCL	-
		AIL	-	-
	ITCL	-	DSCCL, DMCL, DLCL, DLCCL, LCIBL	-
	ALCL	-	LCIBL	-

B- or T-ALL: B- or T-acute lymphoblastic leukemia; B- or T-LBL: B- or T-lymphoblastic lymphoma; B- or T-CLL: B- or T-chronic lymphocytic leukemia; B- or T-SLL: B- or T-small lymphocytic lymphoma; LLI: B-cell lymphocytic lymphoma of intermediate type; LPL: lymphoplasmacytic lymphoma; LGL: large granular lymphocyte lymphoma or leukemia; NK-CLL: NK-cell chronic lymphocytic leukemia; MCL: mantle cell lymphoma; FCCL: follicle center cell lymphoma; MZL: marginal zone lymphoma; NMZ: nodal marginal zone lymphoma; SMZ: splenic marginal zone lymphoma; MALT-L: mucosa-associated lymphoid tissue lymphoma; PCT: plasmacytic tumors; B-LCL: large B-cell lymphoma; DLBCL or DLBCCL: diffuse large B-cell lymphoma, cleaved or not cleaved; LCIBL: large cell immunoblastic lymphoma; CEL: cutaneous epitheliotropic lymphoma; MF/SS: mycosis fungoides/Sezary syndrome; CNEL: cutaneous non-epitheliotropic lymphoma; PTCL: extranodal or peripheral T-cell lymphoma; AIL: angioimmunoblastic lymphoma (also known as angioimmunoblastic lymphomatous disease); ATL: angiotropic lymphoma; ACL: angiocentric lymphoma; AIL: angioinvasive lymphoma; ITCL: intestinal T-cell lymphoma; ALCL: anaplastic large cell lymphoma; Lb: lymphoblastic; Lc: lymphocytic; Cc: centrocytic; Cb: centroblastic; Ib: immunoblastic; Pl: plasmacytic/plasmacytoid; F- or DSCCL: follicular or diffuse small cleaved cell lymphoma; F- or DMCL: follicular or diffuse mixed cell lymphoma; F- or DLCL: follicular or diffuse large cell lymphoma; DLCCL: diffuse large cleaved cell lymphoma; SLLP: small lymphocytic lymphoma plasmocitoid; SNCCL: small non-cleaved cell lymphoma; PCT: plasmacytoma, NOS: not otherwise specified.

**Table 2:** Clinical staging system for canine lymphoma by the World Health Organization (WHO). Adapted from *Withrow and MacEwen’s Small Animal Clinical Oncology* (2013) [1].

Anatomic Site	
**A**	Generalized
B	Alimentary
C	Thymic
D	Skin
E	Leukemia *
F	Others (including solitary renal)
**Stage (to Include Anatomic Site)**	
I	Involvement limited to a single node or lymphoid tissue in a single organ **
II	Involvement of many lymph nodes in a regional area (±tonsils)
III	Generalized lymph node involvement
IV	Liver and/or spleen involvement (±stage III)
V	Manifestation in the blood and involvement of bone marrow and/or other organ systems (±stage I–IV)
**Each Stage Is Subclassified into**	
A	Without systemic signs
B	With systemic signs

* Only blood and bone marrow involved. ** Excluding bone marrow.

**Table 3:** Chemotherapy protocols used in the clinical routine for the treatment of multicentric lymphoma in dogs [1,35,36,37,38,39].

Chemotherapy Protocol	Survival (Days)	Response Time (Days)	Drugs
CHOP+L-asparginase	397	282	Vincristine: 0.5–0.7 mg/m^2^ Cyclophosphamide: 250–300 mg/m^2^ Doxorubicin: 25–30 mg/m^2^ Prednisolone: 2–0.5 mg/kg L-asparginase
CHOP 19 weeks in LDGCB CHOP 19 weeks in PNBCL	325	233	Vincristine: 0.5–0.7 mg/m^2^ Cyclophosphamide: 250–300 mg/m^2^ Doxorubicin: 25–30 mg/m^2^ Prednisolone: 2–0.5 mg/kg
	302	196	
CHOP 15 weeks	311	178	
CHOP 25 weeks in PNBCL	321	209	
Doxorubicin	169	147	25–30 mg/m^2^
CHOP with substitution of cyclophosphamide for lomustine (T and B lymphomas)	291 median 357 B-cell lymphoma 210 T-cell lymphoma	242	Vincristine: 0.5–0.7 mg/m^2^ Lomustine: 40–60 mg/m^2^ Doxorubicin: 25–30 mg/m^2^ Prednisolone: 2–0.5 mg/kg
Procarbazine, Prednisolone and oral Cyclophosphamide	286	197	
LOPP	328	168	Vincristine: 0.5 mg/m^2^ Lomustine: 60 mg/m^2^ Procarbazine: 50 mg/m^2^ Prednisolone: 1 mg/kg
LOPP in T-cell lymphoma	507	431	Vincristine: 0.7 mg/m^2^ Lomustine: 60 mg/m^2^ Procarbazine: 50 mg/m^2^ Prednisolone: 30 mg/m^2^
MOPP	270	189	Mecloretamine: 3 mg/m^2^ Vincristine: 0.7 mg/m^2^ Prednisone: 30–40 mg/m^2^ Procarbazine: 20–50 mg/dog
D-MAC	169	61	Actinomycin D: 0.75 mg/m^2^ Cytosine arabinoside: 200–300 mg/m^2^ Dexamethasone: 0.23 mg/kg Melphalan: 20 mg/m^2^ Melphalan is replaced with chlorambucil after 4 to 6 cycles at 20 mg/m^2^
Tanovea^®^ and doxorubicin	-	194	Rabacfosadine: 1 mg/kg at weeks 0, 6, 12 Doxorubicin: 30 mg/m^2^ at weeks 3, 9, 15
	-	199	Rabacfosadine: 1.0 mg/kg alternated Doxorubicin: 30 mg/m^2^, every 21 days, six total treatments (3 cycles)
Tanovea^®^	-	203	Rabacfosadine: 0.82 mg/kg or 1.0 mg/kg, 30 min, every 21 days, 5 treatments

PNBCL: Peripheral nodal B-cell lymphoma.

**Table 4:** Rescue protocols used for canine multicentric lymphoma [1].

Cycle	Weeks	Drug	Dose and Frequency
1	1	L-Asparaginase	400 U/kg SQ
		CCNU	70 mg/m^2^ PO
		Prednisone	2 mg/kg PO, once daily
	2		1.5 mg/kg PO, once daily
	3		1.0 mg/kg PO, once daily
2	1	L-asparaginase	400 U/kg SQ
		CCNU	70 mg/m^2^ PO
		Prednisone	1.0 mg/kg PO, EOD
	2		
	3		
3–5	1	CCNU	70 mg/m^2^ PO
		Prednisone	1.0 mg/kg PO, EOD
	2		
	3		

SQ: subcutaneous; PO: *per os*, by mouth; EOD: every other day. Note: treatment discontinuation criteria: 1. After conclusion of protocol, two treatments beyond complete response; 2. Progressive disease. 3. Increase in alanine aminotransferase (ALT) activity >2× above normal limit, discontinue drug administration and reinstitution/dose reduction depending on normalization of ALT.

**Table 5:** Prognostic markers and predictive outcomes in canine lymphoma.

Prognostic Factors	Key Insights	Clinical Relevance/Outcome	References
Immunophenotype	B-cell lymphomas have longer remission and survival times than T-cell lymphomas	T-cell lymphomas show shorter remission and survival times compared to B-cell lymphomas	[1,87,88,89]
	CD3+ tumors and B-cell lymphomas with low B5 antigen levels are associated with shorter remission and survival times	Low B5 antigen levels and CD3 positivity predict poor prognosis	
WHO Substage	Substage *b* disease have worse outcomes than those with substage *a* Stages I and II show more favorable outcomes than later stages	Lower substages and earlier disease stages (I and II) are associated with better survival.	[1]
Histologic Grade	Intermediate/high-grade lymphomas respond well to chemotherapy but have a high relapse rate Low-grade lymphomas respond poorly but show longer survival	High-grade lymphomas often relapse early Low-grade lymphomas have longer overall survival	[1]
T-cell Subtypes	Most T-cell lymphomas are aggressive, CD4+/CD45+ high-grade types with low class II MHC expression	High-grade T-cell lymphomas predict poor prognosis	[90]
	A minority of T-cell lymphomas are CD4+/CD45− and show high class II MHC with indolent disease progression	CD4+/CD45− T-cell lymphomas indicate a more favorable, slower-progressing disease course	
Proliferation Assays	BrdU uptake, Ki67 reactivity, and AgNOR indices are used to assess tumor cell proliferation	Higher Ki67 levels predict shorter survival Ki67-low is a positive prognostic marker	[1,91,92]
Molecular Subtypes	CL subtypes include high-grade T-cell lymphomas (LBT, PTCL), low-grade T-cell lymphomas (TZL), and B-cell lymphomas (DLBCL, BL, MZL)	TZLs progress slowly and may respond to low-intensity therapy High-grade T-cell lymphomas are aggressive with poor response	[93]
Biomarkers	TK1, MCP-1, VEGF, MMP, and endostatin have been evaluated in CL Only TK1 showed significant prognostic value Elevated TK1 concentration correlates with cancer presence and shorter survival; combination with CRP enhances predictive power	MCP-1 associated with decreased disease-free interval Elevated serum TK1 is a prognostic marker for disease progression and relapse prediction TK1 + CRP panel could effectively monitor disease and therapeutic response	[14,94,95,96]
Genetic Assay (K9 assay)	TRAF3 mutations are common in B-cell lymphomas, associated with poorer prognosis SETD2 mutation correlates with quicker recurrence SATB1 and FBXW7 mutations in T-cell lymphomas associated with worse prognosis	Specific gene mutations (e.g., TRAF3, SETD2) help predict disease aggressiveness and survival times	[97]
cfDNA	Increased cfDNA levels in cancer patients and correlation with lymph node diameter in multicentric lymphoma	Elevated cfDNA could indicate presence of cancer, predict shorter survival, and reflect response to chemotherapy	[98]
Nucleosome Concentration	Increased in dogs with lymphoma, particularly in B-cell lymphomas and in advanced stages	Elevated nucleosome levels may distinguish between healthy and cancerous states in dogs	[99,100]
	Decrease in remission and rise upon relapse, correlating with disease progression	Reliable biomarker for monitoring disease progression and treatment efficacy	
White blood cell indicators	Increased neutrophil and monocyte counts are related to poorer prognosis in CML treated with CHOP	Elevated neutrophil and monocyte counts predict shorter survival and earlier disease progression	[96,101,102]
	NLR, LMR, and PLR are predictors of time to progression, progression-free survival, and lymphoma-specific survival in canine DLBCL	NLR and LMR correlate with treatment response and survival rates	
	PNR was identified as an independent marker of early lymphoma progression in canine DLBCL treated with CHOP	PNR predicts early progression and time to progression	

BrdU: bromodeoxyuridine; AgNOR: argyrophilic nucleolar organizer region; TK1: thymidine kinase 1; MCP-1: monocyte chemotactic protein-1; VEGF: vascular endothelial growth factor; MMP: matrix metalloproteinase; CRP: C-reactive protein; cfDNA: cell-free DNA; NLR: neutrophil-to-lymphocyte; LMR: lymphocyte-to-monocyte; PLR: platelet-to-lymphocyte.

## Data Availability

Not applicable.
